# Association of age and symptoms with frequency of Premature Ventricular Contractions on 24 hours Holter monitoring

**DOI:** 10.12669/pjms.37.2.2873

**Published:** 2021

**Authors:** Shazadi Ambreen, Humaira Fayyaz Khan, Azmat Hayat, Nasar Abbas Shamsi

**Affiliations:** 1 Dr. Shazadi Ambreen, MBBS, M-Phil Department of Physiology, Shifa Tameere-e-Millat University, Islamabad, Pakistan; 2 Dr. Humaira Fayyaz Khan, MBBS, FCPS Department of Physiology, Riphah International University, Islamabad, Pakistan; 3 Dr. Azmat Hayat, MRCP, FRCP, Fellow Electrophysiology Armed Forces Institute of Cardiology, Rawalpindi, Pakistan; 4 Dr. Nasar Abbas Shamsi, MBBS, M-Phil Department of Physiology, Fauji Foundation University, Islamabad, Pakistan

**Keywords:** BNP, Cardiomyopathy, Dizziness, NT-proBNP, Palpitations

## Abstract

**Objectives:**

This study was done with the objective to identify the determinants of mild, moderate and frequent burdens of premature ventricular contractions (PVCs) which may guide the health care professionals to stratify the high risk patients on basis of their symptoms.

**Methods:**

It was a cross sectional descriptive study conducted in Islamic International Medical College (IIMC) in collaboration with Armed Forces Institute of Cardiology (AFIC) from 18^th^ April 2016 to 20^th^ March 2018. It comprised 60 diagnosed patients of PVCs, divided into three groups on the basis of their PVCs burden determined by Holter monitoring report. Each group of mild (Group-A), moderate (Group-B) and frequent burden (Group-C) constituted 20 patients having PVC burden<10%, 10-20% and >20% respectively. All patients were evaluated for their symptoms by a cardiologist. Statistical analysis was done to determine the association of patient’s symptom and age with mild, moderate and frequent PVCs burden.

**Results:**

PVCs were significantly associated with presence of symptoms as compared to asymptomatic patients. While no significant correlation of age or any specific symptom (palpitations, chest pain, dizziness, shortness of breath) was found with mild, moderate and frequent PVCs burden with p-value of 0.466.

**Conclusions:**

Mild, moderate or frequent PVCs burden are not associated with any specific symptom predominantly or old age. So, it is equally important for all the patients presenting with any symptom of palpitations, chest pain, dizziness or shortness of breath to undergo the work up of PVCs, irrespective of their age.

## INTRODUCTION

Premature ventricular contraction is defined as an extra heartbeat, originating from the ventricles and comes before the normal heart beat.^1^-^3^ Premature Ventricular Contractions (PVCs) are common with an estimated prevalence of 1% to 4% in the general population.^4^

Rapid pacing due to PVCs also results in neurohormonal pathway changes which bring about release of B type neuropeptides (ProBNP). ProBNP is mainly synthesized and secreted by cardiac myocytes in response to myocardial wall stretch.^5^ ProBNP splits into BNP (32 amino acid) and N-Terminal proBNP (76 amino acid).^6^,^7^ Frequent ventricular ectopic beats have a high risk of developing dilated type reversible cardiomyopathy.^8^ A high PVCs burden is an important predisposing factor for developing cardiomyopathy but not every patient with high burden develop cardiomyopathy. Some patients, even with 4% of PVCs burden are reported to develop PVCs induced reversible cardiomyopathy.^5^

The current study was done to identify the most common symptom of PVCs, to correlate the symptoms with different type of PVCs burden and also with age. This study was done with the objective to assess the determinants (such as symptoms, age) for mild, moderate and frequent PVCs burden to guide the physician for identifying frequent PVCs patients which if not treated on time can result in reversible cardiomyopathy. Hence the patients with PVCs can be treated beforehand to prevent its complications.

## METHODS

It was a cross sectional descriptive study conducted in Islamic International Medical College Rawalpindi in collaboration with Electrophysiology department of Armed Forces Institute of Cardiology (AFIC) from 18^th^ April 2016 to 20^th^ March 2018. Ethical review committee (ERC) of Islamic international medical college approved the research protocol (App. #Riphah/ERC/17/0252 issued on 22^nd^ November, 2017). Informed consent was taken from the patients before including in research.

Sixty consecutive patients of PVCs diagnosed on Holter monitoring as mild, moderate and frequent PVCs burden of both gender between 18 to 60 years of age having left ventricular ejection fraction ≥50% were included. Sample size was calculated using W.H.O calculator while prevalence of PVCs was 4%.^2^ PVCs diagnosis was made on 24 hours Holter monitoring report by cardiologist and PVCs burden was calculated by dividing the total premature ventricular beats from total analyzed beats recorded in 24 hours.

All the patients were divided into three groups of A, B and C according to their PVCs burden. Group-A included patients with PVCs burden ≤10%, Group-B comprised patients with PVCs burden10- 20% and Group-C included the patients with PVCs burden≤20%. Cardiovascular status of each patient was evaluated through exercise tolerance test, echocardiography, 24 hours Holter monitoring and blood laboratory data. Information regarding sex, age, height and weight was collected from database.

Patients with ejection fraction less than 50% on echocardiography done by a cardiologist was excluded from the study. Patients with hyperthyroidism and psychological illness like anxiety were also excluded. Finally, all the patients were evaluated for their presenting symptoms in groups A, B and C. Their presenting symptoms such as palpitations, shortness of breath, dizziness and fainting were determined by a cardiologist. In all these patients NT-proBNP levels were also measured by using ELISA Kit (By Elabscience) and statistical analysis was done to assess its association with symptoms of palpitations, shortness of breath, dizziness and chest pain. Chi-square test was applied for categorical variable analysis, ANOVA was applied to compare means of quantitative data and Pearson correlation tests was applied to find correlation of quantitative data. All the statistical analysis was conducted using SPSS version 25 and statistical significance was set at p-value≤0.05.

## RESULTS

### Baseline characteristics

The baseline demographics are listed in Table-I, which shows that more patients presented with symptoms.

**Table-I:** Baseline characteristics of participants.

*Characteristics*		*Group-A (≤10%)*	*Group-B (10%-20%)*	*Group-C (≥20%)*	*p value*	*Total*
Age(mean)		45.9±14.2	47.8±15.9	47.8±17.2	n.s	
Symptoms	Symptomatic	14(23.3%)	17(28.3%)	13(21.7%)	n.s	44(73.3)
	Asymptomatic	6(10%)	3(5%)	7(11.7%)	n.s	16(26.6%)
p value		0.07	0.002*	0.18		0.00*
Gender	Male	10(16.7%)	9(15%)	17(28.3%)		36(60%)
	Female	10(16.7%)	11(18.3%)	3(5%)		24(40%)
p value		1.0	0.65	0.002*		0.12
Total		20	20	20		60

ns: not significant and the data was reported in n and in %.

### Symptoms and PVCs burden

In all burden of less than 10%, 10-20% and more than 20% more patients were symptomatic as compared to asymptomatic patients but the significant difference was found only in Group-B of 10-20% burden with p-value of 0.002 (Table-I). Among all the symptomatic PVCs patients, more patients presented with palpitations and chest pain was the most uncommon complaint but the difference was insignificant (p-value of 0.20 as shown in Table-II).

**Table-II:** Association of symptoms with PVCs burden and NT-proBNP levels.

*Characteristics*	*palpitation*	*chest pain*	*S.O. B*	*Dizziness*	*p-value*
PVCs burden					
Burden <10% (Group-A)	8	1	2	3	
Burden 10-20% (Group-B)	3	3	6	4	0.466
Burden >20% (Group-C)	5	2	3	4	
Total(n)	16(36.4%)	6(13.6%)	11(25%)	11(35%)	0.208
NT-proBNP	0.82±0.43	0.91±0.54	0.87±0.47	0.76±0.58	0.93

In Group-A and Group-C, palpitation was the most common complaint while in burden of Group-B, shortness of breath was the most common complaint but the difference was not significant in either group with p value of 0.46 (Table-II). NT-proBNP levels were measured in all type of complaints and their mean levels suggested no significance difference in any burden (p-value 0.93) as shown in Table-II.

### Age and PVCs burden

There was no significant difference of age among different burdens of PVCs shown in Table-I. No correlation of age was found with premature ventricular contractions with r value of 0.064 as shown in Table-III.

**Table-III:** Correlation of age with PVCs and NT-proBNP levels.

*Age of PVCs patients*		

	*r-value*	*p-value*
NT-proBNP	0.21	0.10
PVCs	0.06	0.62

### Age and NT-proBNP levels

There is no correlation of age with NT-proBNP levels with r value of 0.21 and p-value of 0.10 as shown in Table-III.

## DISCUSSION

The present study demonstrated that PVCs burden is not associated with age, gender or any specific symptom. All the patients of PVCs were almost in the same range in either group suggesting that frequent burden is not necessarily common in old age. No significant association of NT-proBNP and age was found with presence of symptoms as compared to asymptomatic patients. Result of the present study are in contrast to the study conducted by Heart IJC et al which demonstrated the association of old age (61.0±15.2) with PVCs burden of more than 10%.^9^

A population-based study conducted on young healthy subjects, demonstrated the presence of frequent PVCs even in young and healthy subjects. It also demonstrated that frequent PVCs are not only associated with increasing age which is in accordance with finding of present study.^10^

Another study conducted by Ghazala Irfan evaluated the different types of arrhythmia in relation to their presenting complaints. In that study while evaluating the different types of complaints such as Dizziness, palpitations and syncope, the most common symptom in PVCs patients was palpitations.^11^ The present study also showed palpitations as the most common complaint in PVCs patients but the difference was not significant.

Another study demonstrated that asymptomatic patients were associated with high PVCs burden which is contrary to our study which showed no association of symptomatic or asymptomatic patients with High PVCs burden.^12^

Another study demonstrated the association of raised NT-proBNP levels with symptom of fatigue in patients of PVCs in comparison to asymptomatic patients which is contrary to present finding showing no association with any specific symptom.^13^

### Limitations of the study

The study would have been done on a large scale with larger sample size and on multicenter level. It would have been better to measure the myocardial wall stress as well, but due to its unavailability it was not measured.

## CONCLUSION

Frequent PVCs burden are not associated to any specific symptom neither with old age. So, patients presenting with any symptom like palpitations, dizziness, shortness of breath and chest pain should be evaluated for premature ventricular contractions irrespective of their age.

### Authors’ Contribution:

**SA:** Conceived, designed, data collection, manuscript writing and is responsible for integrity of work.

**HFK, AH:** Drafting the work and revising it critically for important intellectual content.

**NAS:** Analysis and interpretation of the data for work.
